# The naked truth about HIV and risk taking in Swedish prisons: A qualitative study

**DOI:** 10.1371/journal.pone.0182237

**Published:** 2017-07-31

**Authors:** Sigrid J. A. Lindbom, Markus Larsson, Anette Agardh

**Affiliations:** Social Medicine and Global Health, Department of Clinical Sciences, Lund University, Malmö, Sweden; University of North Carolina at Chapel Hill, UNITED STATES

## Abstract

**Background:**

This qualitative study explores former prison inmates’ perceptions and attitudes towards HIV risk inside Swedish prisons.

**Method:**

In 2014, eight semi-structured interviews were conducted with former male prisoners to gain a deeper understanding of situations perceived to be associated with risk of HIV transmission. The material gathered from the interviews was analyzed by manifest and latent qualitative content analysis.

**Results:**

The findings revealed that risky behavioral practices, such as sharing needles, unprotected sexual activity, and lack of openness about HIV status represented potential health threats with regard to the risk of HIV transmission.

**Conclusions:**

Evidence from the study indicates that educational interventions regarding HIV and the transmission routes are required for HIV prevention in Swedish prisons.

## Introduction

Current evidence indicates that prisons are high-risk environments for the transmission of the Human Immunodeficiency Virus, HIV [1–3]. These high-risk environments are created by the high prevalence of HIV positive inmates, lack of HIV prevention strategies, most notably needle exchange programs and accessibility of condoms, and extreme social conditions [1]. Although most HIV infections are contracted prior to incarceration rather than in the prison, individuals who circulate between incarceration and the community are at high risk for transmitting and contracting HIV [4]. Individuals in the penal system are often disenfranchised with considerable vulnerabilities, such as poor education, unemployment, and limited access to health care, thus increasing the likelihood of HIV risk-taking behavior occurring before, during, and after incarceration [5].

Risky behavior, including needle sharing, sharing equipment for tattooing or piercing and unprotected sex, may facilitate intra-prison HIV transmission [6]. Prevalence of injective drug use (IDU) in prisons has been shown to vary from 2.4% to 74%, depending upon the setting [2]. In one study from Australia, 46% of the 355 participants with a history of IDU continued to inject drugs while imprisoned and in another Australian study 39.2% of the 735 prisoners reported having injected drugs in the prison [7, 8]. Studies have shown that although injective drug usage diminishes during incarceration, needles are shared more frequently [9]. Piercing and tattooing constitute risk for HIV transmission since unsterile equipment is often used, which increases the risk of HIV transmission [10]. Tattooing in prisons may be common; studies documenting this have found that between 33% and 60% of the study population had their tattoo made while incarcerated [10–13]. One study from Australia reported that 27% of those 67 respondents who had been tattooed in the prison shared a needle for this purpose [12]. Moreover, inmates who have prison tattoos made are significantly more likely to have a history of injective drug use [12, 14]. Due to the multiple HIV risk factors that prisoners are exposed to, it would be difficult to estimate the risk for HIV transmission that might be attributable to tattooing. However, the combination of the high prevalence of tattooing and inmates with blood borne infections makes tattooing in prisons a risky practice for HIV transmission [5].

Studies from various countries have further documented that sexual activity between inmates and between inmates and visitors occurs on a regular basis in prisons [3, 15–19]. In an Australian survey of 2018 male prisoners, 7.1% reported consensual sexual contact with another male inmate and a study in the US found that 36% of the 174 male prisoners had engaged in oral sex with another internee [18, 20]. Furthermore, it has been estimated that around 13% of inmates in American prisons have been sexually victimized during incarceration [21]. In a study of 6964 male prisoners in the US, Wolff an colleagues found that 4.8% had been victims of sexual assaults while serving their punishment [22]. While non-consensual sexual activities such as rape and sexual coercion carry a higher risk of HIV transmission, the lack of condoms and lubricants in prisons may also contribute to heightened risk of HIV transmission even in consensual sexual activities between inmates but also during conjugal visits [3, 16, 23]. In Ohio, US, 7.4% and 3.9% of the 916 male prisoners surveyed reported consensual vaginal- and anal intercourse, respectively, and 15.4% reported condom use [17]. However, information regarding sexual activity between male inmates may be difficult to obtain due to fear of homophobic violence and methodological and ethical challenges related to such research [3]. Moreover, many men who have sex in prison do not identify themselves as homosexual and might not have had sex with other men before they were imprisoned, and hence might be less inclined to report such behaviors [2, 3]. All in all, efforts to gain reliable knowledge about consensual and non-consensual sexual activity in prison environments remain somewhat of a challenge [24, 25].

The HIV prevalence in Swedish prisons is 5% compared with the national prevalence of 0.06% [26]. The main contributing factor to the high HIV prevalence in prisons is the high proportion of people with injective drug use [27]. However, Swedish research exploring risks of intra-prison HIV transmission is scarce. A study from European prisons, representing only one prison in Sweden, showed that 25% of the 135 participants from Sweden had ever injected drugs, and of those individuals 67% had injected inside the prison [27]. Almost a third of the Swedish participants had had sexual intercourse with a female while in prison, but only 7% had used a condom. Tattooing was reported by 29%, and the only reported methods for cleaning the equipment were water and/or heat [27]. In Sweden, prison culture has been described as a homophobic environment where violence and threats are made against homosexual prisoners, who are viewed as having the same low status as those convicted of rape or pedophilia [28].

Although more research is clearly needed on HIV risk-taking behavior among Swedish prisoners, practitioners and policy makers with pragmatic concerns about harm reduction might benefit from research that seeks to gain a deeper understanding of how inmates themselves experience and evaluate HIV risks in prison environments. In this regard, qualitative research methods have been especially helpful for exploring inmates’ perceptions, attitudes, and behavioral intentions [29, 30]. Thus, Razzaghi and colleagues emphasized the need for understanding how risk behaviors are actually practiced, in order to generate hypotheses for future research and prevention strategies [31]. Moreover, research based on prisoners’ own narratives may be preferable to complex and costly surveys, due to the difficulties in obtaining reliable quantitative data regarding illegal behavior such as illicit drug use [31].

Nevertheless, there are significant barriers to undertaking qualitative research inside the prison setting. The hierarchical and coercive nature of the prison environment may pose a hinder for establishing a trusting and confidential interview climate. Also, the emotional state of the inmates, due to the frustrating and depriving prison environment, can result in erroneous conclusions from data gathered through interviews carried out in prisons [32]. Therefore, in order to overcome some of these challenges, the current qualitative study sought to interview former inmates in the community rather than while they were incarcerated.

The overall aim of this qualitative study was thus to gain a deeper understanding of former inmates’ experiences of HIV inside Swedish prisons. More specifically, the study sought to understand former inmates’ views concerning situations that they believed to be associated with risk of HIV transmission. A study exploring risk-taking behaviors in Swedish prisons may not only help to address knowledge gaps and encourage further research, but also has the potential to advocate for HIV prevention strategies directed towards a vulnerable, marginalized, and risk-taking population. Successful HIV prevention strategies have to take the needs and living conditions of the targeted population into account.

## Method

### Study setting

The present study was conducted in Scania, a province in the south of Sweden. This province was selected primarily for administrative purposes, to avoid extensive travel costs related to conducting the interviews. Sweden has in total 46 prisons, and these are classified into three security levels where level 1 has the highest security level and 3 the lowest. The Swedish Prison and Probation Service is the responsible government agency for the prison system and decides where in Sweden the inmate will serve their sentence depending on the conviction, risk of flight, and need of treatment, among other factors [33]. The facility should also be able to offer some type of activity that corresponds with the inmate’s needs. Around 93% of all prisoners in Sweden are men and the major reason for incarceration 2014 was crime of violence and drug offense [34, 35]. In 2014, there were 4133 persons admitted into a prison facility (detention excluded) [36].

### Study participants

The study was limited to males above the age of 18, who had been imprisoned anywhere in Sweden during the last 10 years and were living in the region of Scania at the time of the study.

Study participants were identified through national interest organizations working with former inmates. The organizations disseminated an introduction letter to their members, describing the background and purpose of the study, as well as contact details to the researcher (SL). Men who were interested then contacted the researcher, and a time and a place of convenience for the participant’s interview was agreed upon. In total, eight former inmates were interviewed during a three-month period in 2014.

### Study procedure

Qualitative research methods were used in order to gain a deeper understanding of the participants’ experiences of risks that could lead to HIV transmission inside the prisons. Eight in-depth, semi-structured face-to-face interviews were performed, using open-ended questions and participants were given the opportunity to freely elaborate upon their answers. The interviews were conducted at a private location. Before the interview began, the first author (SL) explained the purpose, that participation was fully voluntary, that their information would be kept confidential, and that the participants could refuse to answer a question or interrupt the interview at any time. To address any potential discomfort that the questions could cause, participants were provided with information about where to turn if they had questions or needed support regarding sexual health and HIV. Furthermore, permission to record the interview was also sought. However, we did intentionally refrained from collecting personal or detailed demographic background data, in order to preserve anonymity and privacy in a vulnerable group. Verbal, rather than written, consent was sought in order to protect participants’ confidentiality. All participants gave their informed consent verbally before the interview started.

A thematic interview guide was used, which included questions that addressed perceptions of the risk of acquiring HIV in the prison, and experiences of drugs, tattooing, sexual activities, HIV testing and access to prevention programs in the prison. The interviews took approximately one hour, and no remuneration was given for participation. After eight interviews, there was a common understanding among the co-authors that saturation had been reached as no new information continued to emerge.

### Data analysis

Interviews were conducted in Swedish, recorded, and transcribed verbatim by the first author (SL) into Swedish for analysis. The material was analyzed using manifest and latent content analysis according to Graneheim and Lundman [37]. The interview was considered the unit of analysis. The transcribed interviews were reread several times by the first author in order to gain an overview of the material. Thereafter, meaning units were identified and shortened into condensed meaning units, which were coded. Subsequently, codes with similar contents were subsumed into categories, representing the manifest content. The categories were then reviewed by the other co-authors (ML, AA), who provided feedback. This step enabled comparison of similarities and differences between the categories and facilitated the identification of themes, i.e. the latent content of the text. Three overarching themes were identified and agreed upon by the authors. When the analytical model was finalized, the results were translated into English. An example of the coding procedure is shown in Table 1.

**Table 1:** An example of the coding procedure.

Meaning Unit	Condensed Meaning Unit	Code	Category	Theme
I did not need to keep track of when drugs entered the department. I knew since I had a long queue outside my cell when people wanted to borrow the needle.	When drugs entered. I had long queue outside my cell when people wanted to borrow the needle.	Queue for my needle	Injection with shared needles	*Queuing for the needle*
And when a girl came in, it might sound disgusting, but they were supposed to, they are swallowers most of them that come in. Do you understand what I mean? These girls, that is what it is supposed to be, that is the sport, they should be swallowers.	When a girl came in, might sound disgusting, but they are swallowers most of them, that is the sport.	The girls are swallowers	Women like men like us	*Sex according to the norm*

The study was approved by the Regional Ethical Review Board in Lund (ref. no. 2014/57).

## Results

Eight in-depth interviews were conducted with males who had previous experience of the Swedish prison system. Four of the participants had experience from several different prison facilities and the accumulated time of incarceration ranged between 1–17 years (see Table 2 for participants’ experiences of the prison system). Although no longer incarcerated, some of the recruited participants still had restrictions at the time of the interview, for example, residing in a drug therapy facility or only released during the day. One participant had immigrant background. Most of the participants were former drug users, and the majority had been involved in injective drug use.

**Table 2:** Participants’ experience of the Swedish prison system.

Participant	Total time spent in prison (for all sentences)	Number of prisons incarcerated at	Time elapsed since release
1	17 years	5	8 months
2	1.5 years	2	4.5 years
3	Incarcerated 2 times, time unknown	1	4 years
4	Incarcerated 9 times, time unknown	15	13 months
5	Incarcerated 24 times, time unknown	1	7 years
6	10 years	1	2 years
7	4 years	3	4.5 years
8	1 year	1	Released on condition

Three themes emerged from the analysis of the interviews; *Queuing for the needle*, *Sex according to the norm* and *Needing a HIV protection strategy*. Each theme with its respective categories is represented in the analytical model shown in Table 3. The findings are presented in the following sections and structured according to the analytical model; themes are in bold and categories are italicized.

**Table 3:** Overview of analytical model: From codes to themes.

Codes	Categories	Themes
Needle sharing common		
No needles meant sharing	Injection with shared needles	
Queue for my needle		
Can ask about Hepatitis when sharing		
Hepatitis more common than HIV	HIV in the shadow of hepatitis	Queuing for the needle
Everyone has Hepatitis		
Shared tools for tattooing		
Only one needle for tattooing	Unaware of risks with tattooing	
Did not think about risks with tattooing		
Girls want to visit criminals		
Visits from “volunteers”	Women like men like us	
The girls are swallowers		
HIV future problem		
Did not think about risk with HIV	Disregarding sex as a risk for transmission in the heat of the moment	Sex according to the norm
HIV not relevant for several years		
Light years away from American films		
Homosexuality does not exist	Gay sex and rape–this is not America	
People do not like gay people		
HIV+ should be separate	“HIV people” should be separated	
Cautious around HIV+		
Fighting creates HIV anxiety		
Do not know who has HIV	Identifying potentially risky situations	Needing a HIV protection strategy
Avoiding toilets and food		
Risk with HIV not discussed		
No one said anything so you say nothing	Lacking information about HIV	
Received no info about HIV		

### Queuing for the needle

Former inmates reflected upon their experiences of drug usage in the prison environment and expressed that most risk-taking behaviors were motivated by a desire to inject drugs. Participants were aware of the risks of HIV associated with needle sharing but the social stigma surrounding HIV prevented an open discussion between inmates regarding the infection and its transmission routes.

*Injection with shared needles*. The thought of becoming infected through injective drug use was suppressed consciously by an overriding need to inject. It was stated that “I knew what I got myself into” (Participant 3) and that it is “a lottery” (Participant 3). Injective drug use occurred frequently inside the prison, and participants had personal experiences of sharing needles or witnessing other inmates doing it. One participant explained: “you would rather have a needle and no amphetamine then the other way around” (Participant 4). Access to needles and syringes was reported to be very limited, resulting in that inmates had to share needles in order to inject drugs.

*”Yes*, *you shared them*. *Because there was no access to needles and syringes*.*”* (Participant 5)

The scarcity of available needles created a demand for the needles available among inmates with an addiction. Accounts revealed that queues of up to 40 people were formed outside the cell of inmates who possessed a needle, once drugs had arrived inside the prison.

*“I did not need to keep track of when drugs entered the department*. *I knew since I had a long queue outside my cell when people wanted to borrow the needle*. *And*, *there you have in some way the naked truth about needles and needle sharing inside prisons*.*”* (Participant 4)

The lack of access to clean needles inside Swedish prisons was regarded as a problem. It was suggested that clean needles should be provided inside prisons, and not having them was “crazy”, since it was the only way to reduce the risk of HIV transmission. However, it was also expressed that it would be “unreasonable” or “impossible” to expect that the Swedish Prison and Probation Service should provide clean needles, given the drug free policy inside Swedish prisons.

The lack of the possibility to clean existing needles inside the prisons was also highlighted. Boiling syringes or using boiled water to clean the needle were mentioned as methods of reducing the risk of HIV infection.

*“There is a lot of sharing needles*. *But they boil them you know*, *they put them in a pot with boiling water and boil them so it is quite low risk to be infected then I believe*. *It is more if they rinse it under the tap; you do not see any blood but there is still some blood left*. *I believe people get infected that way*.*”* (Participant 1)

*HIV in the shadow of hepatitis*. Participants spoke vividly about Hepatitis C, and the interviews revealed that inmates tended to assume that other prisoners, who also injected drugs, had hepatitis due to its high prevalence in this population:

*“If you are*, *or have been*, *an injective drug user*, *then you have Hepatitis C*.*”* (Participant 6)

The expectation that other injective drug users also were infected with hepatitis had normalized the discourse around the disease and inmates could discuss the issue before sharing injection devices:

*”You ask the question ‘Have you*…*You also have C* [Hepatitis C- author’s remark] *right’? You can ask that question*.*”* (Participant 5)

However, when it came to HIV, attitudes differed:

*“In some way you assume that*, *if he has HIV he will say*. *Maybe*. *Yes*. *You never ask*.*”* (Participant 3)

Even in instances when inmates were uncertain about their own HIV status, the possibility of HIV infection was surrounded by silence:

*“I knew I had Hepatitis C*, *I knew that*, *and I had known that for a long time*, *for 20 years*. *And like… no I did not know if I had HIV*. *But like*, *you only said you had Hepatitis C*.*”* (Participant 5)

Participants speculated about whether the silence around HIV was related to a fear of enduring scorn and discrimination if one were to disclose a positive or even uncertain HIV status. Inmates with HIV infection, it was thought, would be “outcasts and thrown out of the department” (Participant 6).

*Unaware of risks with tattooing*. Inmates disregarded risks associated with tattooing as means of transmitting HIV–even when tools such as homemade machines were shared for this purpose. Participants reported that inmates seemed to be unaware about the infection risks associated with having tattoos made with only one needle available:

*”I did not*, *I did not think about the risk*, *I did not think at all about it…with the needle and such things*. *Luckily I did not do it*, *but there were many that had tattoos made in there*. *And I do not think they were aware of that it was such a risk*.*”* (Participant 7)

However, the experiences of tattooing inside the prison varied, and some participants had never heard or seen anything related to tattooing among inmates.

### Sex according to the norm

Sexual activities during incarceration were exclusively perceived in heterosexual terms. This contributed to an omnipresence of heterosexual norms in the prison and left limited space for sexual activities outside this norm.

*Women want men like us*. Sexual activities between inmates and visitors were described as common, occurring on a daily basis, and”if you have visitors of course you have sex” was a typical expression. Visitors could be girlfriends or partners but also women who were friends with one of the other internees or paid to visit an inmate in exchange for sex. Another category of women mentioned was women who actively approached inmates for sexual relations. Inmates felt that the criminal status itself was a means of attracting women. This was illustrated by the observation that when an inmate was released from prison, his woman would start visiting another inmate:

*“Well*, *one girl used to visit one of the inmates*. *When he got released from the prison she came and visited someone else*. *Girls are so*… *well they sometimes find it exciting with people like us*.*”* (Participant 7)

During the interviews, the concept of “swallowers” emerged, referring to visitors who swallowed the semen after giving oral sex:

*“And when a girl came in*, *it might sound disgusting*, *but they were supposed to*, *they are swallowers most of them that come in*. *Do you understand what I mean? These girls*, *that is what it is supposed to be*, *that is the sport*, *they should be swallowers*.*”* (Participant 2)

However, for those with a permanent partner outside the prison, sexual contacts with visitors were of little relevance and rarely observed.

*Disregarding sex as a risk for transmission in the heat of the moment*. Risks related to unprotected heterosexual sex, such as HIV transmission, were described as something inmates did not think about in general, at least not in the situation. The lack of sex inside the prison contributed to inmates being less cautious when it came to engaging in unprotected sex:

*“In there we are all*, *all …*.*us men are more or less starved for it* [sex–author’s remark]. *When you have been doing time for two years and not had a girl you will kind of take anything*, *right? Then*, *anything becomes beautiful*. *Even a pig in bikini becomes totally wonderful*.*”* (Participant 2)

However, anxiety could appear after the sexual encounter and distress inmates:

*“We do not think there* [in the prison- author’s remark], *we do not*. *Afterwards you do*. *Like it is afterwards*, *then you may have ruined the rest of your life*, *kind of*.*”* (Participant 3)

Participants had mixed experiences of the availability of condoms inside the prisons. For some, condoms were described as completely absent, also in the visiting rooms. If condoms were going to be used, women had to bring these when visiting inmates. Others had the opposite experience; condoms had always been available, for example in a bowl in the visiting room or available to the inmates before entering the visiting room.

*Gay sex and rape–this is not America*. Sexual activities between inmates were described as non-existent, never seen, heard of or experienced inside the prison. Homosexuality in prison was sensitive and labeled as taboo. If it existed, it was extremely rare since it was unlikely that two people “like that” would find each other in a strictly controlled prison environment:

*“Homosexuality does not exist in Swedish prisons*. *I can say that during all the years I have spent in prison*, *I have never encountered it anywhere*. *Never*. *So it is out of the question for me*.*”* (Participant 1)

The prison culture was described as macho with heterosexual views and values–an environment that marginalized gay people and made it impossible to be open about a sexual orientation deviant from the pervasive heterosexual norm:

*“I do not believe people tell*, *because there are a lot of people who do not like gay people for example*. *Or pedophiles or those who abuse women and children*, *they all have the lowest status in prison*.*”* (Participant 3)

Participants typically expressed that “I am not gay, so I don’t know anything about that”, that everyone had visitors so there was no need “for that” and that you “never want to experience that it exists”. Any homosexual elements inside the prison were strictly humorous.

Sexual violence between inmates was perceived as non-existent, far removed from what was described as “the situation in America”, or in American films. Sexual violence was regarded as unaccepted and when it was referred to, it was usually in the form of urban myths:

*“Here in Sweden*, *it is taboo with homosexuality in prison*. *It is not as in America where they will rape the first young person that comes in because it is ‘young flesh’*…*”* (Participant 2)

Others had heard about rape in prisons, reading about it in the newspaper or hearing about it from other inmates:

*“I heard about a young guy that had been raped by several inmates*, *and after that*, *he could not tell the correctional officers because he would be subjected to further threats*. *It could have led to his death*, *maybe*.*”* (Participant 7)

### Needing a HIV protection strategy

When reflecting on the risk of HIV infection inside the prison, inmates described feelings that were both complex and sometimes contradictory. Uncertainty about other inmates’ HIV status and transmission routes of the virus created both anxiety and frustration directed towards other inmates and the authorities, and a need for taking protective measures.

*“HIV people” should be separated*. Participants believed that the presence of HIV positive inmates increased the risk of becoming infected. Inmates should therefore be informed when a HIV positive internee arrived in their prison department, so that the other inmates could be aware of the risks and be extra careful around him. As a protective measure, it was suggested that prisoners living with HIV should be separated from the other inmates:

*“Then maybe they should open a special department for that kind of people* [people living with HIV- author’s remark] *that expose others to risks*. *I mean*, *I have been convicted*, *okay I was convicted for something and I will serve my punishment*. *Should I then also in here*, *expose myself to a risk that will accompany me for the rest of my life?”* (Participant 7)

*Identifying potentially risky situations*. Due to the incarceration, inmates’ privacy was limited and participants described that the physical contact with other prisoners sometimes resulted in concerns regarding HIV transmission. A typical example of a situation that was considered risky was when fights between inmates occurred, resulting in exposure to blood:

*“Then he came into my cell and started to hit me and I hit him back and then he was bleeding in the nose and I was bleeding here in my hand*. *I found out afterwards that he has HIV and I exploded with anger because the staff in there* [the prison- author’s remark] *had not said anything*.*”* (Participant 6)

Participants described a psychological distress that persisted several months after the incidents where a fight had erupted with a HIV positive inmate. There was a feeling that the Swedish Prison and Probation Service had the ultimate responsibility and could avert these situations by informing the inmates when a HIV positive internee arrived.

Sharing of communal items, such as glass, cutlery, plates, showers, toilets, food, and clothes was believed to be a risk factor for HIV transmission. Concerns were expressed regarding the fact that inmates with HIV used the same items as everyone else.

*”When a HIV positive person does the dishes he could cut himself on a glass*, *or something*, *and then puts the glass back and someone else comes and takes it and drinks directly from it*. *The glass is still warm and so the blood has not solidified*.*”* (Participant 2)

When discussing cooking, inmates did not think that those with HIV should work in the kitchen due to the risk of transmitting the disease:

*“You should not work in the kitchen if you have hepatitis or HIV*. *It is so easy to cut yourself; even though you are wearing gloves it is so easy*.*”* (Participant 6)

Toilets were also described as a potential source of HIV transmission in particular, as in some prisons there were very few facilities. Concerns were expressed regarding the possibility that HIV could be transmitted through feces:

*“When I was in the prison*, *sitting with eh…people from yes*, *from the East*: *Russia*, *the Baltics and Africa*, *whom I more or less shared toilet with–sure the thought occurred to me that*, *well I am not an expert on blood infections*, *but there was a fear there*, *it was*.*”* (Participant 8)

When showering, inmates took extra precautions to avoid blood from the previous user, who could have left blood from shaving or wounds on the foot:

*“Before showering I always poured as hot water as possible in the coffee maker and brought it with me to the shower and splashed it around before I stepped inside since maybe someone could have dripped blood or so*. *Many people cut themselves there and it dropped there on the floor*, *and I do not know what disease they have so I went around thinking about it all the time*.*”* (Participant 2)

*Lacking information about HIV*. HIV was, accordingly, not discussed openly in the prison. Despite anxiety over HIV, a discussion between inmates was absent:

*”…I went around thinking about it all the time*, *but no one else said anything*, *so then you do not say anything*.*”* (Participant 2)

Furthermore, the discussion about HIV was not only absent among inmates, participants reported that prison authorities failed to provide information about HIV:

*“I have never received any information from the Swedish Prison and Probation Service about risks with diseases and such things*. *There was never any talk about that*, *I have never experienced that in prison*.*”* (Participant 3)

The absence of HIV information was problematized, and participants emphasized that such information was crucial in order to prevent risk-taking and promote earlier HIV testing among inmates. When reflecting on HIV testing, some inmates could not recall that a test had been offered while incarcerated. Other inmates recalled that when offered testing for HIV and hepatitis, the staff had been insensitive and had not explained the implications of the test results:

*“And when she* [the nurse- author’s remark] *came to report the test results*, *well she told me something like this `You don’t have HIV and you have Hepatitis C but you already knew that´ she said and then*, *and then she left*.*”* (Participant 4)

## Discussion

To our knowledge, this is the first qualitative study exploring inmates’ experiences of HIV in the Swedish prison environment. The overall results from this study show that the participants experienced risk-taking behavior, such as sharing of needles and unprotected sexual activities that could lead to the transmission of HIV while inside the prison. Drug use appeared to be the primary driver for ignoring risk-taking when sharing needles with other inmates. At the same time, concerns were raised regarding the risks of HIV transmission in combination with injecting drugs, and the interviews revealed that fear and anxiety of being infected with HIV while imprisoned were prevalent. Moreover, former prisoners varied in their perceptions of the extent that HIV transmission risk was present and the particular types of situations that increased such risk.

The participants expressed that HIV transmission through sexual activities between inmates, both voluntary and forced, was non-existent and taboo. This view has been corroborated by previous research in Sweden, which has highlighted that homosexuality is controversial and taboo in prisons [28]. However, the possibility exists that participants in this study experienced or knew of same-sex sexual relationships but were unable to mention this in the interview situation due to the prevailing stigma around homosexuality in prisons. Previous research shows that it is difficult to obtain information about sexual activities between men in prisons [2].

The accounts from this study suggest that there is very limited discussion around HIV inside the prison. While the normalization of Hepatitis C facilitated own disclosure, little or nothing was mentioned about HIV. There are two plausible reasons for this. The first is that injecting inmates were either confirmed HIV negative or believed that they were so. The HIV incidence among injecting drug users in Sweden has indeed been declining since 2013, in part due to more people in this population being on anti-retroviral treatment [38]. The other reason concerns the stigma that very often surrounds HIV, which hinders people from talking about it. Such stigma may be particularly problematic for containing the infection inside the prison, if fear of repercussions from other inmates prevents prisoners from being tested [39].

Yet, anxiety over possible HIV infection was a recurring theme, also for those participants who had not been involved in injective drug use. The interviews revealed a lack of general knowledge about HIV and the transmission routes, which created unnecessary high levels of fear and anxiety and might also exacerbate discriminatory practices against people living with HIV. People who are living with HIV experience discrimination across all societal domains, and in the prison they risk being targeted by other inmates [40]. Implementing educational HIV programs for prisoners therefore appears to be a crucial aspect in alleviating fears about HIV and reducing stigma and discrimination. Both classroom and peer-based educational programs have proven to be successful in increasing HIV knowledge and ameliorating prejudices among inmates [41, 42]. Grinstead and colleagues found in a comparison study from the US that inmates preferred having HIV messages delivered by a peer who was living with HIV than by a professional HIV educator [43]. Such an approach may also have promising effects in decreasing HIV discrimination since it encourages contact and interaction with inmates who are living with HIV, thereby targeting the silence around the infection.

Prisoners are especially vulnerable to violation of their human rights due the fact that their liberty has been removed. The World Health Organization highlights that inmates should have the same right to health care, including HIV prevention programs, as those living in the community [44]. Prevention programs can include distributing condoms, needle exchange programs (NEP), actions to prevent sexual abuse, substitute therapy, HIV education, voluntary testing, treatment, and support [3]. However, there is often a lack of condoms or sterile needles in prisons [45]. Prison systems and governments have argued that NEPs cannot be introduced due to safety issues. According to Jürgens et al. [2], implementing such programs would mean that the prison authorities have to acknowledge that they have failed.

The participants in this study did not always experience equal rights to health while imprisoned. Residents in southern Sweden can readily access a NEP but no prison in Sweden currently participates in a needle-exchange program. However, research shows no negative consequences in prisons where a NEP has been implemented [15]. NEPs have been introduced in over 60 prisons in 11 countries with no evidence of increased drug use or increased security concerns in those prisons [3, 45].

While prisons are described as high-risk environments for HIV transmission by for example the World Health Organization [46], evidence from this study suggests that there are gaps in how the Swedish Prison and Probation Service provides information about HIV and HIV testing and that some prison facilities fail to offer condoms. This is problematic since inmates tend to have a history of high-risk behaviors before incarceration, such as unprotected sexual intercourse, and drug and alcohol abuse, i.e., risk factors that are related to HIV [39]. The potential for HIV prevention in Swedish prisons could be hindered by prison officers’ attitudes. A survey carried out in southern Sweden revealed that 38% of the prison officers did not believe that condoms should be available to inmates [47]. Reasons that were given mainly revolved around the idea that sexual relations should not be encouraged in prisons. Thus, it might be crucial to target prison staff with HIV education in order to facilitate HIV prevention programs in prisons.

The risky practices described by these former Swedish prisoners in relation to injective drug use, tattooing, and sexual behavior while inside prison generally concur with finding from previous studies concerning prisoners in a variety of international settings [10, 13, 27, 30]. More evidence-based knowledge about HIV risk in Swedish prisons is needed. In this respect, both qualitative and quantitative methods would be warranted in order to gain a comprehensive understanding of the HIV infection risk and transmission routes among inmates.

### Trustworthiness of the study

In an effort to enhance the credibility of the study, examples of the coding process have been provided, and quotes have been used throughout the text to ground the findings in the participants’ own words. Moreover, participants were located through a variety of different channels and lived in different geographical locations in the south of Sweden, increasing the likelihood that different backgrounds and experiences were represented. The participants had extensive experiences from prisons in Sweden, and altogether they had spent several decades in prisons all over Sweden, which further enhanced the credibility of the findings. Some participants had experiences from more than one correctional facility, including up to 15 different prisons nationwide, which adds to the transferability of the results. To reach confirmability, the researcher bracketed any pre-understandings and consulted with the co-authors during the analytic process. Dependability was achieved by discussion of the coding procedure between the co-authors in order to arrive at consensus.

A number of limitations in this study may have had an impact on the results. The length of imprisonment varied among the participants, ranging from one year to a decade or more in prison. Hence, the experiences might vary over time, with some experiences more recent in time and others more distant. However, all participants had been in prison within the last 10 years and several of them had been recently released, i.e. within the past two years. Almost all of the participants in this study were former addicts, and some had been injective drug users. However, at the time of the interview, all reported that they were drug-free and had left the criminal life. Inmates who have no experience of IDU or who are currently in prison might have different experiences from the participants in this study. Moreover, only one participant had immigrant background. Although saturation was reached by the eighth interview, it cannot be excluded that recruitment of additional participants might potentially have led to new information. Even though efforts were made to ensure that the participants felt safe and comfortable during the interview situation, the sensitive nature of the topic, and the stigma and taboo surrounding it could have hindered them from speaking openly and freely about their experiences. It is possible that the fact that the interviewer was a woman could have influenced the participants’ willingness to share sensitive information.

Finally, the purpose of the current study was to explore former inmates’ perceptions regarding HIV risk in the prison environment, in order to understand to what extent harm reduction efforts might be needed in this particular setting. The extent to which HIV risk assessments and risk-taking decisions made during periods of imprisonment continued to influence the participants’ risk-taking behavior after they had been released into the community is entirely unknown, and further studies would be needed to shed light on this important aspect.

## Conclusions

The main study finding indicating that prisoners experienced risky behaviors such as needle sharing and engaging in unsafe sexual activities, suggest that the Swedish prison population is an important target group for HIV prevention. In light of these findings, it is important to reinforce the HIV prevention programs inside Swedish prisons. Preventive measures could include introducing needle exchange programs, making condoms easily available and offering opt-in HIV testing. Moreover, educational efforts, directed at both inmates and staff, are required to prevent the spread of HIV, diminish unnecessary fear and anxiety, decrease the stigmatization of HIV positive inmates, and to facilitate a positive climate towards preventive programs among the staff and the inmates. However, further research about the factors that may contribute to risk-taking behaviors is needed to gain a better understanding of the HIV infection risk inside Swedish prisons.

## Supporting information

S1 Text Informed consent form in English. (PDF)

S2 Text Interview guide in English. (PDF)

S3 Text Informed consent form in Swedish. (PDF)

S4 Text Interview guide in Swedish. (PDF)
