# Supplementary material for: The naked truth about HIV and risk taking in Swedish prisons: A qualitative study
Source: PLoS One. 2017 Jul 31;12(7):e0182237. doi: 10.1371/journal.pone.0182237 (PMC5536296; doi:10.1371/journal.pone.0182237)
Supplement: S1 Text — (PDF) [file pone.0182237.s001.pdf]

LUNDS UNIVERSITET
Medicinska fakulteten

Malmö, 2014-01-22

Interviews about former inmates perceptions of risk exposure to HIV during incarceration

You have received this letter since you have showed interest in participating in a study about men's experiences of risk of exposure to HIV during incarceration. All men who have been in prison during the last 10 years and are living in the region of Scania are welcome to participate in the study, regardless of HIV status. Everything that is said during the interviews is dealt with completely anonymous. By investigating whether inmates experienced risks, prevention measures can be envisaged to prevent the spread of HIV.

Knowledge about whether inmates are exposed to risks that could lead to an HIV infection in Swedish prisons is very limited. Statistics show a higher incidence of HIV (5%) among the prison population compared with the general population (0.06%). To prevent the spread of HIV in Swedish prisons, we need more knowledge about the prisoners' experiences out of risk situations that may arise.

I will, as part of my degree at Master level, conduct interviews to gather more information about the experiences of risk situations that may involve exposure to HIV. The results from the study will be presented in the form of a thesis. A simplified report in Swedish will also be made available.

Participation is voluntary and anonymous. No names or personal details of those included in the study will be collected through the interviews. All information will be presented at a group level so that no individual can be identified. For instance, I do not write the names of the prisons where you have been incarcerated. It is possible for those who participate in the study to withdraw from and terminate the interview at any time.

Participating in the study means that you attend an interview, which is expected to take approximately 45 minutes. During the interview, a tape recorder will be used if you consent. The material from the tape recorder will be transcribed and the files will then be deleted. The transcribed material will be analyzed to compile a final thesis and a report. During this process, only me and my supervisor at the Social Medicine and Global Health at Lund University will have access to the material.

If you have any questions about the study, please feel free to contact me.

Kind regards,

Sigrid Lindbom
Student,
Lund University

fha12sli@student.lu.se

Dr. Anette Agardh
Research manager,
Lund University
040-39 13 38
anette.agardh@med.lu.se

Do you have questions about sexual risk or HIV and want to talk to someone?
Please contact Lena Söderquist, psychologist at Noaks Ark Syd, who has extensive experience of working with issues related to HIV and sexual health. You can remain anonymous. Email: lena.soderquist@noaksark.org.
