# Supplementary material for: The naked truth about HIV and risk taking in Swedish prisons: A qualitative study
Source: PLoS One. 2017 Jul 31;12(7):e0182237. doi: 10.1371/journal.pone.0182237 (PMC5536296; doi:10.1371/journal.pone.0182237)
Supplement: S2 Text — (PDF) [file pone.0182237.s002.pdf]

Interview guide

Themes that were discussed:

- What are your thoughts about the risk of contracting HIV in prison?
- Can you tell me a about your experiences of the presence of drugs in prison?
- What is your experience of tattooing and body piercing in prison?
- What are your experiences regarding whether inmates had sexual contacts?
- Are condoms available in prison?
- What are your experiences whether prisoners thought about the risk of contracting HIV?
- Did you experience the prison environment as a vulnerable environment for HIV?
- What do you think can be done to prevent the spread of infection?
- Do you have something you would like to add?

Background Information
When and how long have you been in prison?
Which prison and safety level were you situated at?
