# Supplementary material for: The naked truth about HIV and risk taking in Swedish prisons: A qualitative study
Source: PLoS One. 2017 Jul 31;12(7):e0182237. doi: 10.1371/journal.pone.0182237 (PMC5536296; doi:10.1371/journal.pone.0182237)
Supplement: S3 Text — (PDF) [file pone.0182237.s003.pdf]

LUNDS UNIVERSITET
Medicinska fakulteten

Malmö, 2014-02-21

Intervjuer om före detta intagna mäns upplevelser av riskutsatthet för hiv under fängelsetiden

Du har fått detta brev då du visat intresse att delta i en intervjustudie som handlar om mäns erfarenheter av risker att utsättas för hiv under fängelsetiden. Alla män som har suttit i fängelse de senaste 10 åren och är bosatta i Skåne är välkomna att delta i studien, oavsett hiv status. Allt som sägs under intervjuerna behandlas helt anonymt. Genom att undersöka om intagna upplevt risker kan förebyggande insatser planeras för att förhindra spridningen av hiv.

Kunskapen kring interners riskutsatthet för hiv på svenska fängelser är i dagsläget mycket begränsad. De studier som gjorts visar på en högre förekomst utav hiv (5%) bland interner jämfört med den generella befolkningen (0,06%). För att förhindra spridning utav hiv på svenska fängelser behöver vi mer kunskap kring interners erfarenheter utav risksituationer som kan uppstå.

Jag kommer, som del i mitt examensarbete på mastersnivå, att genomföra intervjuer för att samla in mer information kring erfarenheter av risksituationer som kan innebära utsatthet för hiv. Studiens resultat kommer sedan att presenteras i form av en uppsats. En förenklad rapport med resultaten kommer även att görs tillgänglig.

Deltagandet är frivilligt och anonymt. Inga namn eller personuppgifter på de som ingår i studien kommer att samlas in genom intervjuerna. All information kommer att presenteras på gruppnivå så att ingen enskild individ kan identifieras. Vi kommer till exempel inte skriva vilken anstalt du har befunnit dig på. Det är möjligt för de som deltar i studien att närsomhelst dra sig ur och avsluta intervjun.

Deltagandet i studien innebär att du deltar i en intervju, vilket beräknas ta cirka 45 minuter. Under intervjun används en bandspelare om du godkänt detta. Materialet från bandspelaren kommer att transkriberas och filerna kommer sedan raderas. Materialet kommer att analyseras för att ställa samman en examensuppsats, en rapport samt publiceras i internationella vetenskapliga tidsskrifter. Under denna process kommer endast jag och min handledare vid Socialmedicin och global hälsa på Lunds Universitet att ha tillgång till materialet.

Har du några frågor om studien är du naturligtvis välkommen att höra av dig till undertecknad.

Hälsningar,

Sigrid Lindbom
Studerande,
Lunds Universitet
0736-42 68 12
fha12sli@student.lu.se

Dr. Anette Agardh
Forskningsledare,
Lunds Universitet
040-39 13 38
anette.agardh@med.lu.se

Har du frågor kring sexuella risker eller hiv och vill prata med någon?
Kontakta Lena Söderquist, leg. psykolog på Noaks Ark Syd som har lång erfarenhet av att arbeta med frågor kring hiv och sexuell hälsa. Du kan vara anonym. Tel: 0704-32 92 33, e-post: lena.soderquist@noaksark.org.
