# Supplementary material for: The naked truth about HIV and risk taking in Swedish prisons: A qualitative study
Source: PLoS One. 2017 Jul 31;12(7):e0182237. doi: 10.1371/journal.pone.0182237 (PMC5536296; doi:10.1371/journal.pone.0182237)
Supplement: S4 Text — (PDF) [file pone.0182237.s004.pdf]

Intervjuguide

Teman som kommer diskuteras:

- Kan du berätta om dina tankar kring risken att smittas av hiv i fängelse?
- Kan du berätta lite om dina erfarenheter av förekomst av droger i fängelset?
- Vad är dina erfarenheter av tatuering och piercing i fängelset?
- Vad är dina erfarenheter kring om intagna hade sexuella kontakter?
- Var kondomer tillgängliga på fängelset?
- Vad är dina upplevelser av om intagna funderade på risken att smittas av hiv?
- Upplevde du fängelsemiljön som en utsatt miljö för hiv?
- Vad tror du kan göras för att förhindra smittspridning?
- Har du något du skulle vilja lägga till?

Bakgrundsinformation
När och hur länge har du suttit i fängelse?
Vilken anstalt och säkerhetsklass befann du dig på?
